# Supplementary figures and images for: MYC disrupts transcriptional and metabolic circadian oscillations in cancer and promotes enhanced biosynthesis
Source: PLoS Genet. 2023 Aug 28;19(8):e1010904. doi: 10.1371/journal.pgen.1010904 (PMC10491404; doi:10.1371/journal.pgen.1010904)

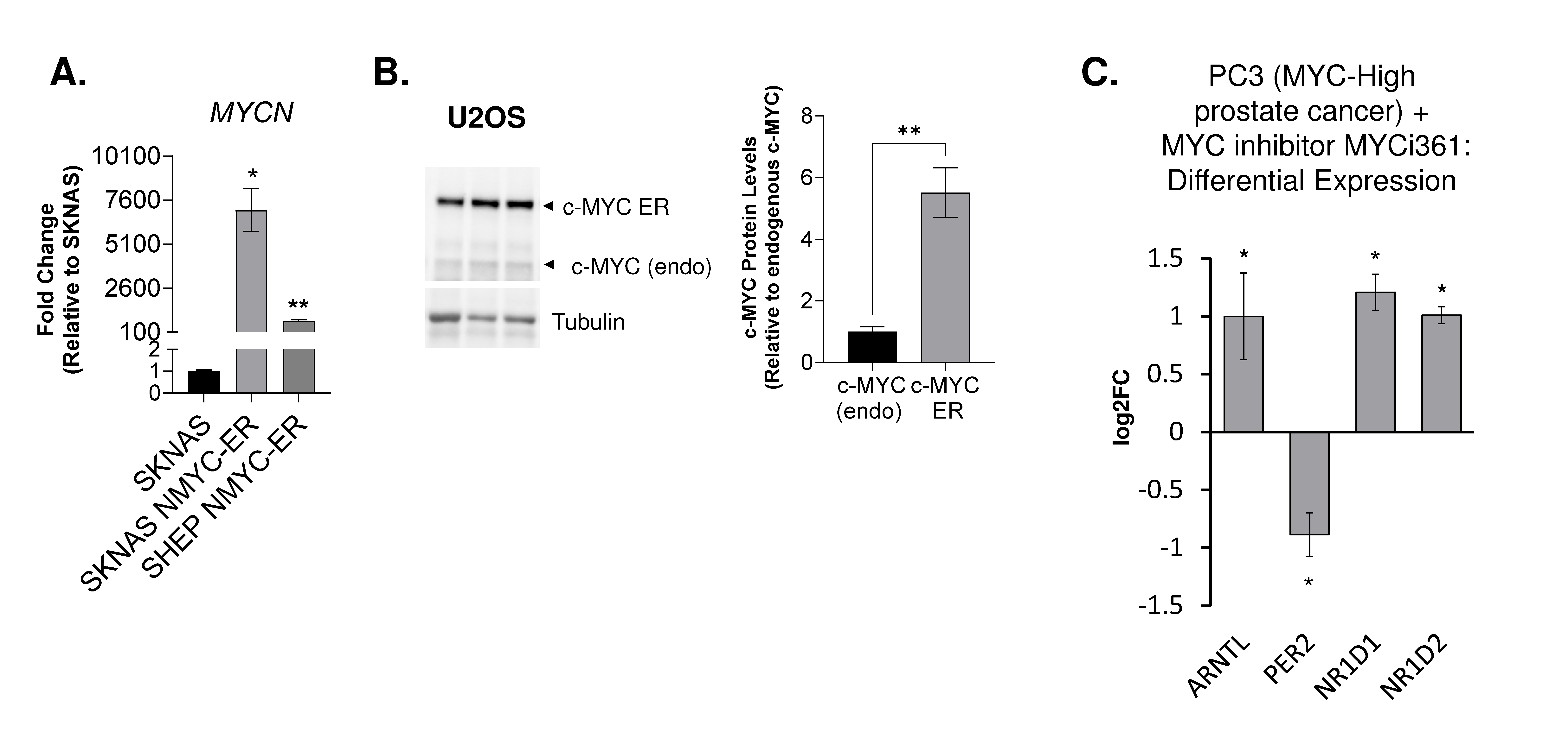

Supplement: S1 Fig — A. MYCN was determined in parental SKNAS, SKNAS N-MYC-ER, or SHEP N-MYC-ER by qPCR in n = 3 replicates by quantitative PCR (qPCR), normalized to β2M. MYCN overexpression is shown relative to parental SKNAS, since SHEP do not express MYCN. B. C-MYC-ER or endogenous c-MYC (endo) are shown in n = 3 immunoblot replicates, and quantitation is shown in the right panel, relative to Tubulin. C. The PC3 prostate cancer cell line, known to express high MYC, was treated with 6 μM of the MYC inhibitor MYCi361 for 24 hours in biological triplicates, as previously published [41]. Raw RNA-sequencing data was downloaded and processed (see Methods), and Deseq2 was used to compare cells ± MYCi361. Log2FC of indicated genes is shown. For A,B, error bars are standard error of the mean (S.E.M.) and ** is p < 0.01 and * is p < 0.05 by Welch’s Corrected Student’s T-test. For C, error bars are Standard Error as calculated by DeSeq2, and * indicates padj < 0.005. (TIFF) [file pgen.1010904.s002.tiff]

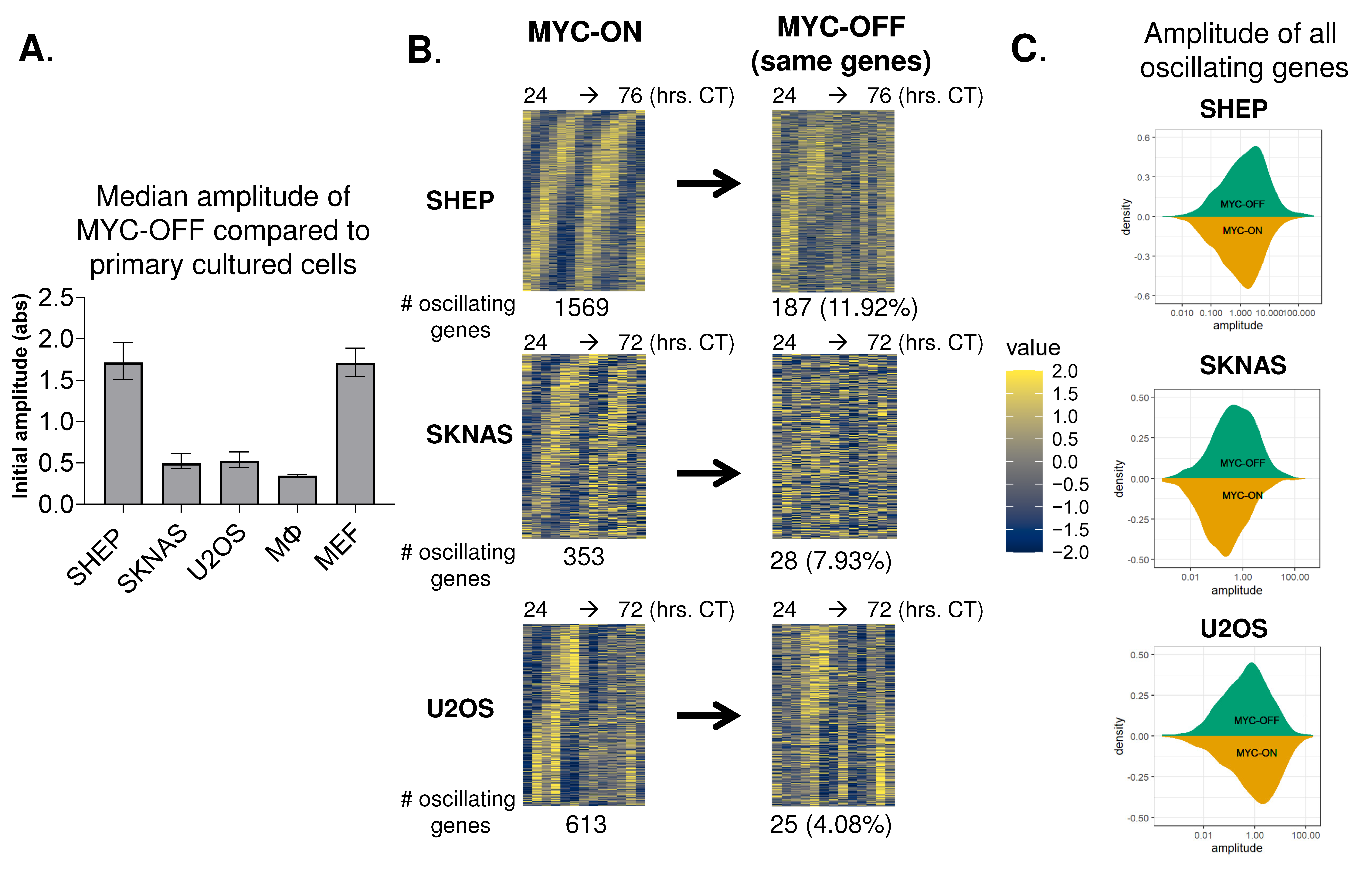

Supplement: S2 Fig — A. The median initial amplitude (absolute, abs) of oscillating genes in SHEP, SKNAS, and U2OS MYC-OFF, as determined by ECHO and shown in Fig 2, was compared to previously published ECHO analysis of time-series RNA-sequencing of entrained primary macrophages (n = 3 biological replicates) or new ECHO analysis of previously published and downloaded time-series RNA-sequencing of entrained primary mouse embryonic fibroblasts (n = 4 biological replicates, see Methods) [9,43]. Error bars represent 95% confidence intervals. B. RNA-sequencing was performed on SHEP N-MYC-ER, SKNAS-N-MYC-ER or U2OS MYC-ER ± 4OHT and + dexamethasone, with RNA samples collected every 2–4 hours at the indicated timepoints. RNA was analyzed for rhythmicity by ECHO for both MYC-OFF and MYC-ON, with genes with a 20–28 hour period and with BH.Adj.P.Value < 0.05 deemed to be rhythmic. These genes were sorted by phase and are presented in a heatmap for MYC-ON. For MYC-OFF, the same genes that are rhythmic in MYC-OFF are presented in the same order, but with MYC-OFF values instead. N = 2 time series were used for each cell line. C. The amplitude of oscillation of each gene in MYC-OFF and MYC-ON, as determined by ECHO, was graphed as a mirrored density plot to allow direct comparison between each condition, with MYC-OFF on the top and MYC-ON on the bottom. (TIFF) [file pgen.1010904.s003.tiff]

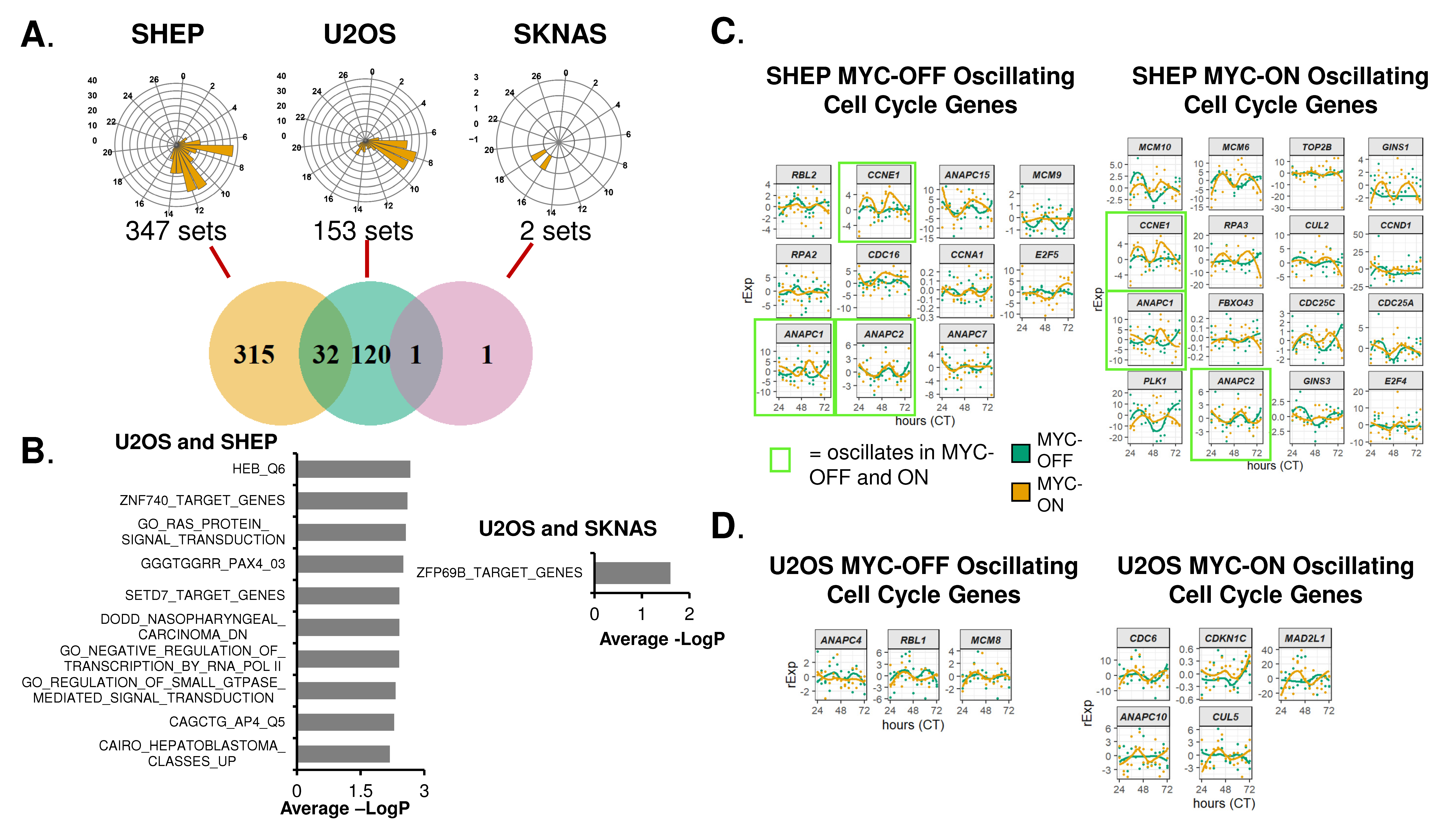

Supplement: S3 Fig — A. The pathways deemed to be significantly oscillatory in MYC-ON cells by PSEA (20–28 hr period, q-value (vs. background) < 0.2 and p-value (vs. background) <0.1) for each cell line were binned and graphed on a polar histogram. The scale for each histogram is on the left side. Overlap of oscillatory programs by Venn diagram is also shown. B. The identity of oscillatory programs in MYC-ON cells from (A) are plotted by average of–LogP from each cell line of overlap. The most highly significant pathways (up to 10) for each overlap are shown. C,D. Cell cycle genes deemed to be oscillating by ECHO for MYC-OFF and MYC-ON SHEP (C) and U2OS (D) are graphed, with dots representing the RNA-sequencing TPM values, and lines indicating the fitted oscillation curves as calculated by ECHO. Genes with a green border are oscillatory in both MYC-OFF and MYC-ON. (TIFF) [file pgen.1010904.s004.tiff]

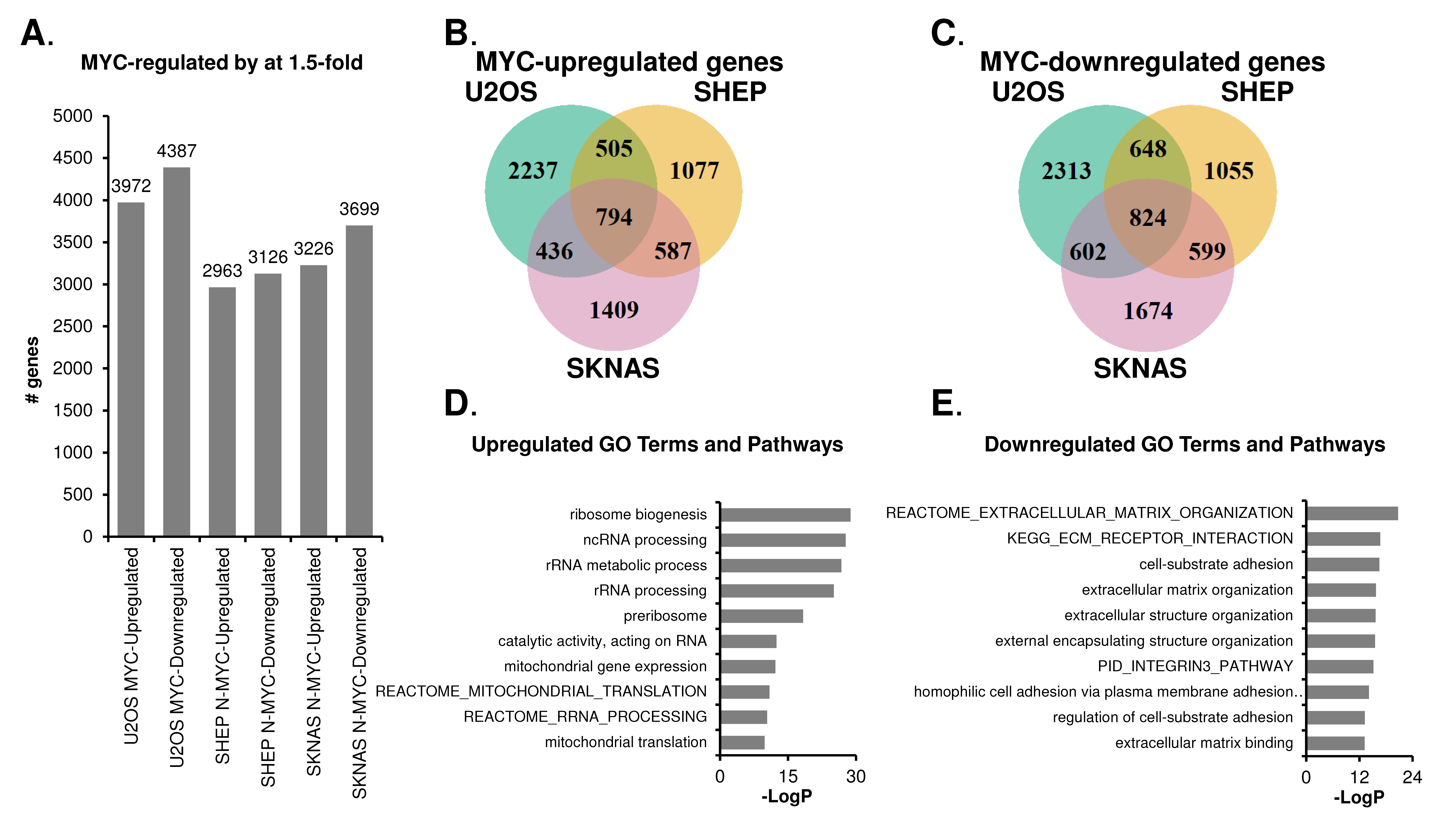

Supplement: S4 Fig — A. Differential expression analysis, using DeSeq2, was performed on MYC-ON vs MYC-OFF for SHEP N-MYC-ER, SKNAS-N-MYC-ER or U2OS MYC-ER, with p.adj < 0.05 deemed significant. Genes that were up- or down-regulated in MYC-ON by at least 1.5-fold are shown. B,C. Venn diagram of the overlap of 1.5-fold upregulated (B) and downregulated (C) genes by MYC. D, E. Genes that were upregulated or downregulated in all 3 cell lines were subjected to pathway analysis with the ToppFun suite, and pathways with FDR B&H < 0.05 were deemed significant. For upregulated (D) and downregulated (E) genes, the most highly enriched pathways from the Pathway and GO libraries are shown in the table, ranked by–LogP. (TIFF) [file pgen.1010904.s005.tiff]

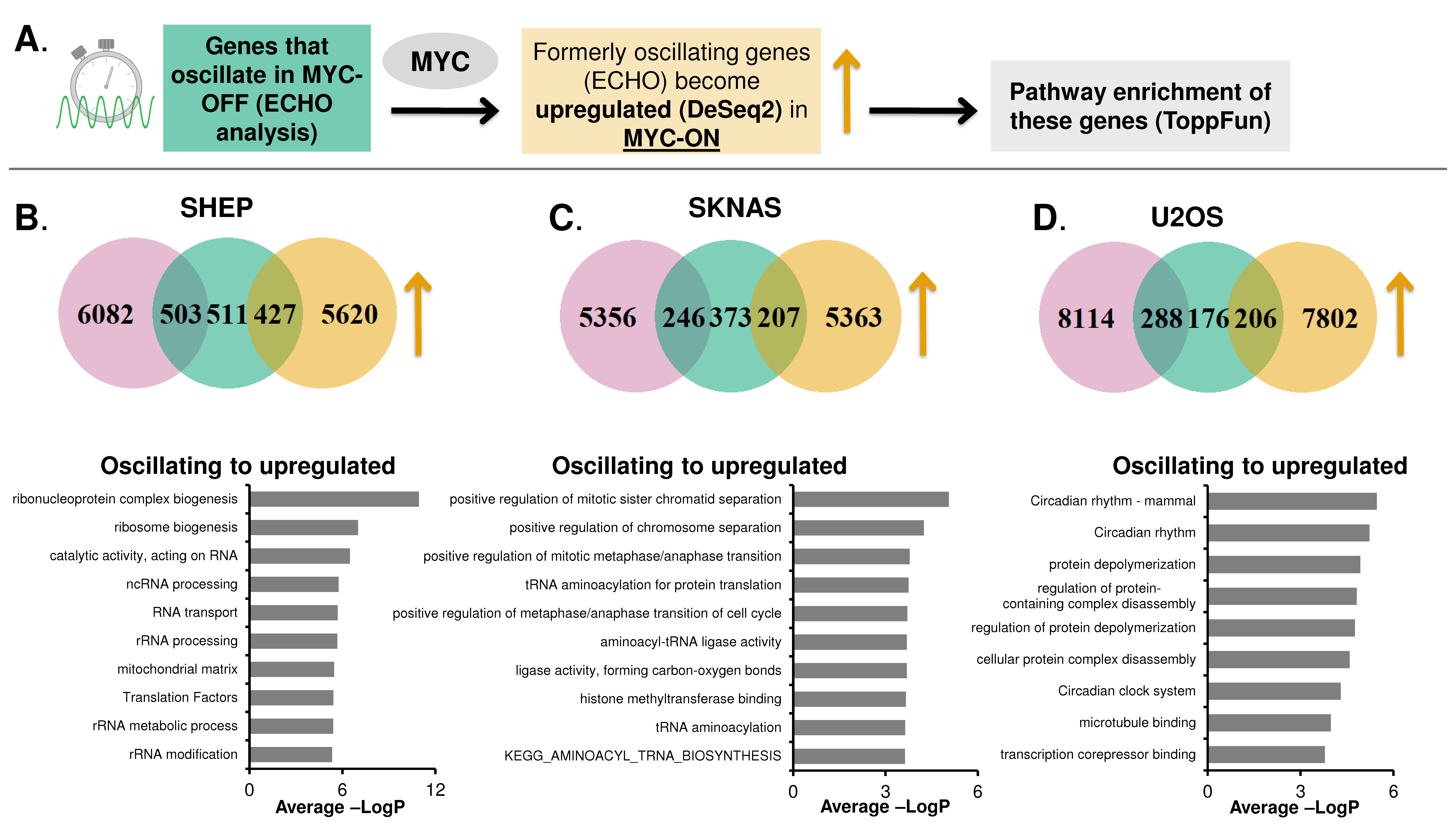

Supplement: S5 Fig — A. Illustration of workflow to identify genes that lose oscillation when MYC is activated (by ECHO), which of these genes become up- or down-regulated by MYC (using DeSeq2), and analysis of these genes for pathway enrichment by ToppFun. B-D. Venn diagram from SHEP N-N-MYC-ER (B), SKNAS N-MYC-ER (C), or U2OS MYC-ER (D) of genes (identified by ECHO) that were circadian in MYC-OFF (center, green), and lost oscillation and became either downregulated (left, pink) or upregulated (right, tan) in MYC-ON (identified by DeSeq2). Bottom section shows the most highly enriched upregulated pathways in each cell line, as identified by ToppFun enrichment from the Pathway and GO libraries of genes that lost oscillation and became upregulated. Pathways are ranked by–LogP, and FDR B&H < 0.05 for all pathways. For SKNAS, we focused on output pathways; full results, including the most enriched pathways that correspond to the molecular circadian clock are available at Figshare, doi: 10.6084/m9.figshare.23729373.. (TIFF) [file pgen.1010904.s006.tiff]

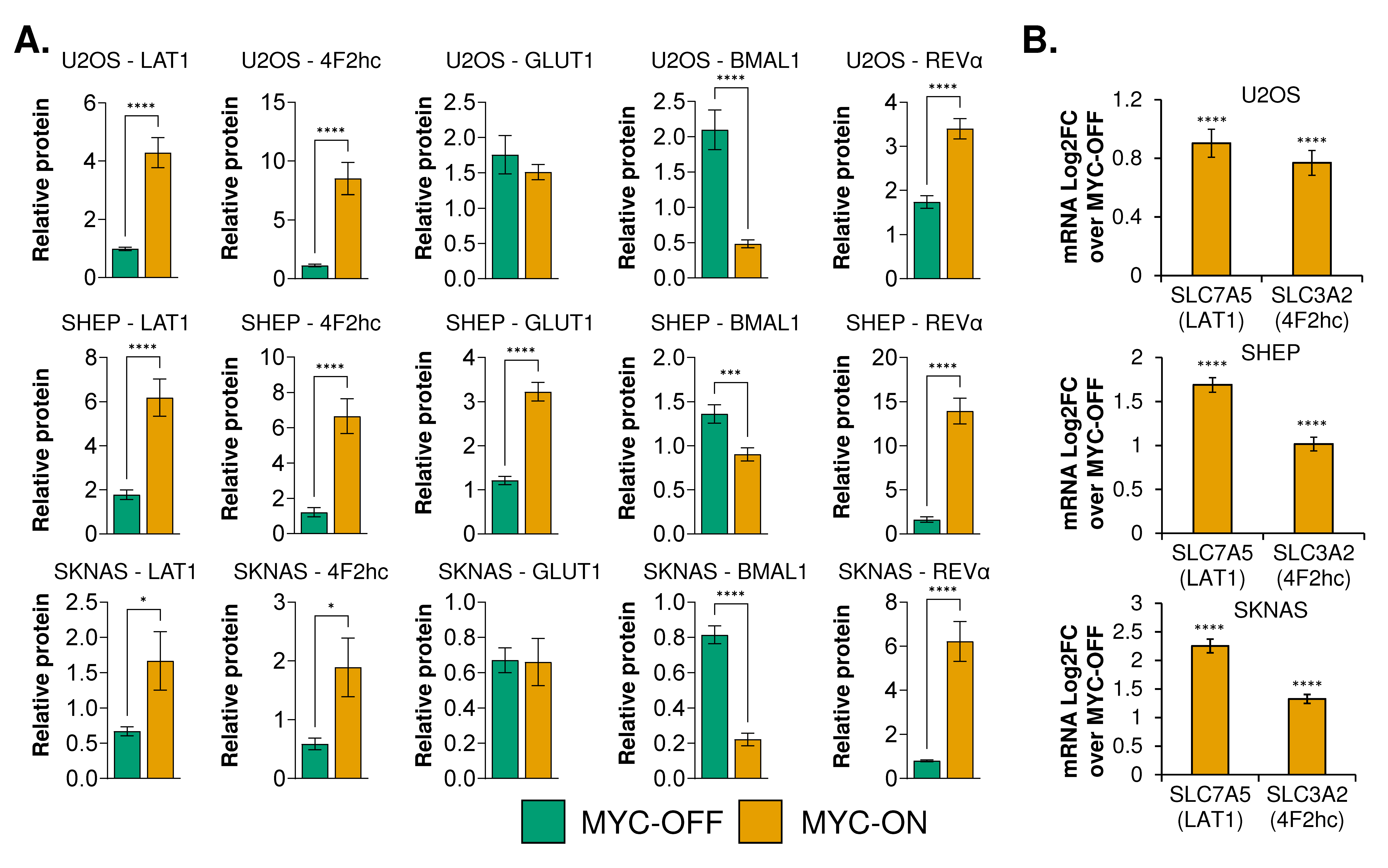

Supplement: S6 Fig — A. Immunoblots in Fig 6 were quantified, relative to Tubulin, for each indicated protein, and MYC-ON was compared to MYC-OFF for each band and each replicate regardless of time. B. Log2 Fold Change in mRNA expression levels, from DeSeq2 analysis of RNA sequencing, of SLC7A5 (LAT1) and SLC3A2 (4F2HC) in U2OS MYC-ER, SHEP N-MYC-ER and in SKNAS MYC-ER under MYC-ON (4OHT) compared with MYC-OFF (Ethanol) condition. For (A), error bars are S.E.M, and for (B), error bars are standard error of Log2FC as calculated by DeSeq2. For both panels, **** is p < 0.0001, *** is p < 0.001, and * is p < 0.05 by Welch’s corrected Student’s T test (A) and adjusted P value from DeSeq2 (B). (TIFF) [file pgen.1010904.s007.tiff]

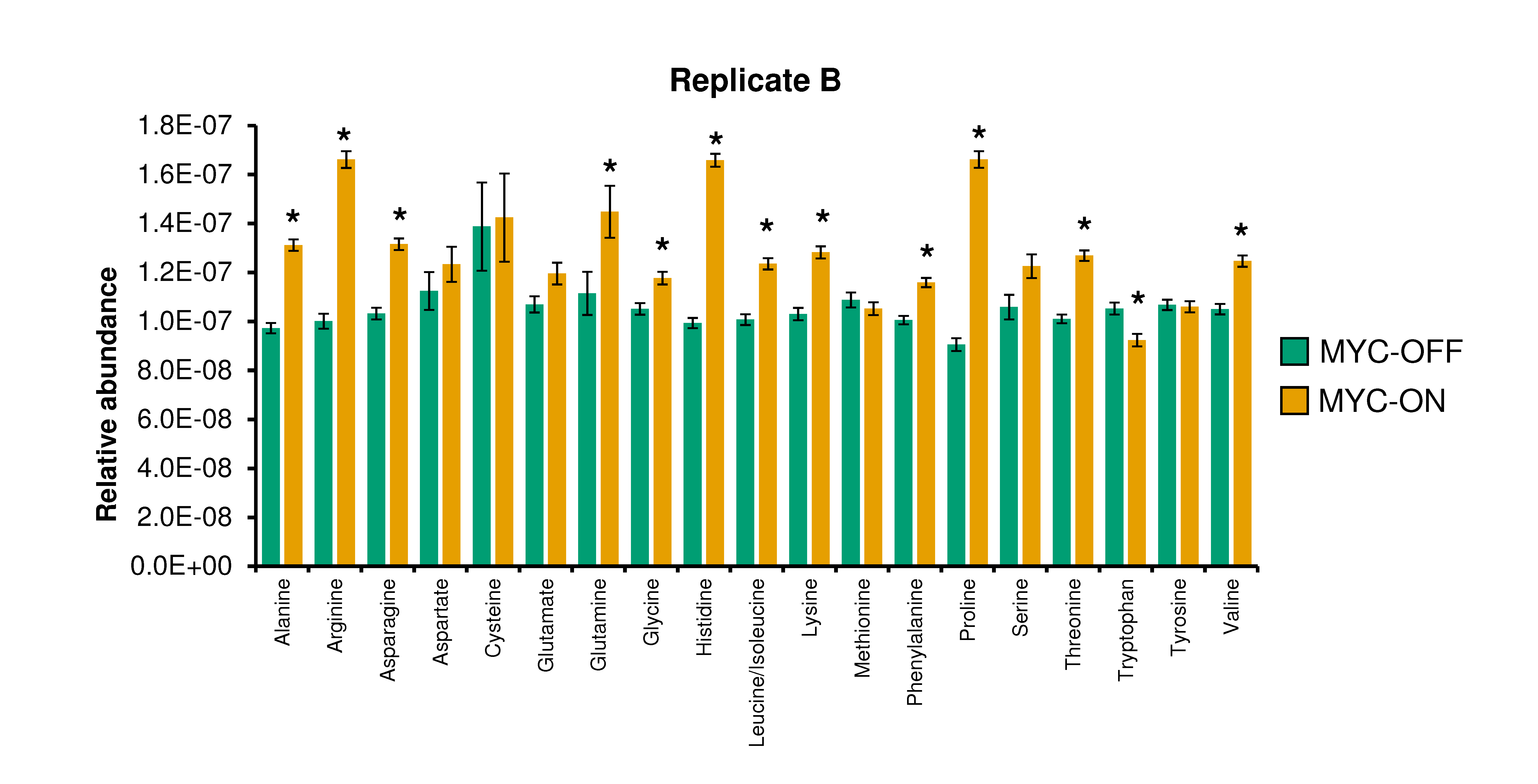

Supplement: S7 Fig — A replicate experiment (Replicate B) of Fig 7C was performed. LC-Mass spectrometry was performed on U2OS MYC-ER treated ± 4OHT for at least 48 hours. N = 25 circadian timepoints for MYC-OFF and MYC-ON were averaged as biological replicates, normalized to cell number for each collection. **** is p < 0.0001, *** is p < 0.001, ** is p < 0.01, and * is p < 0.05 by Welch’s corrected Student’s T test. (TIFF) [file pgen.1010904.s008.tiff]

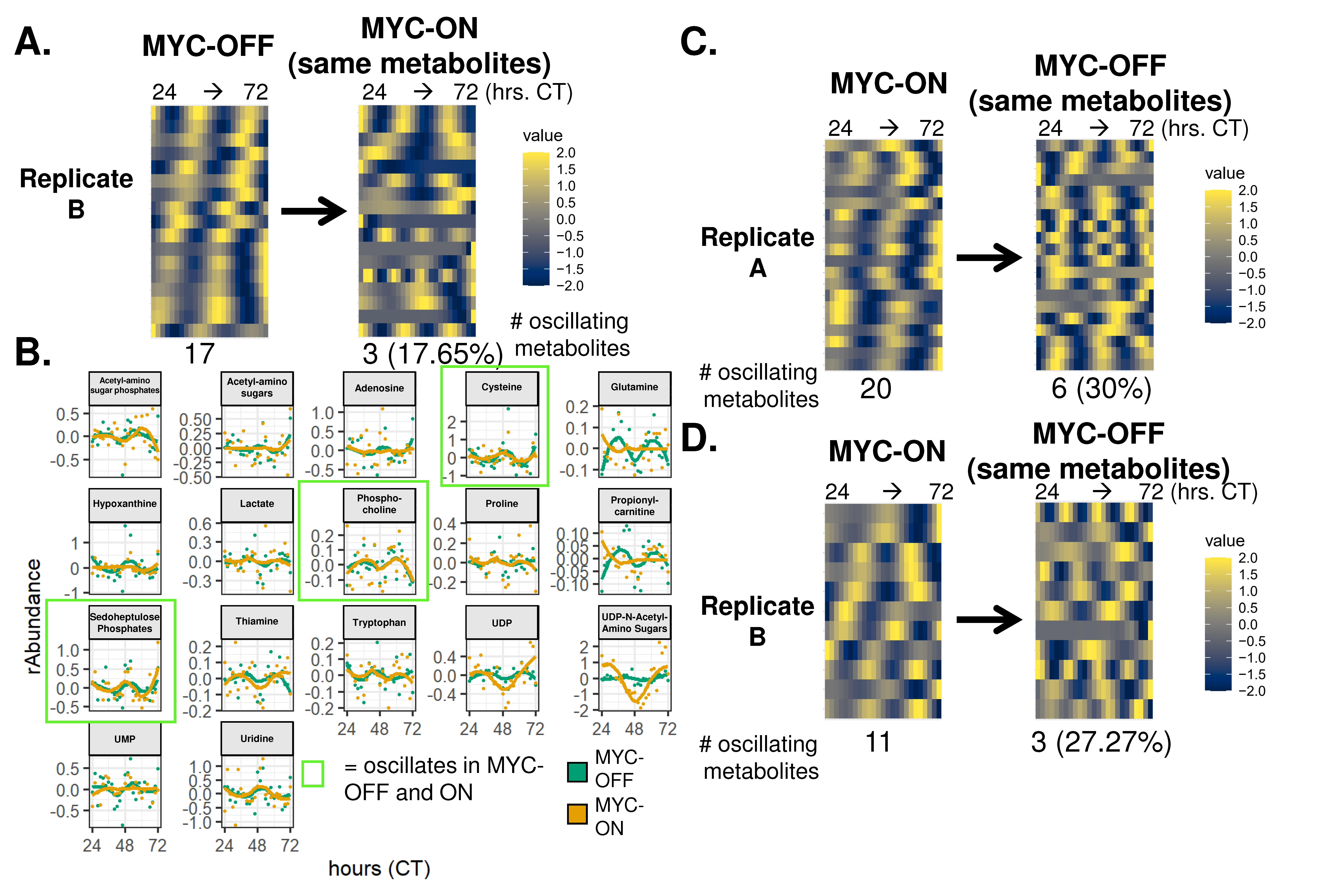

Supplement: S8 Fig — A. An independent replicate (‘Replicate B’) of time-series metabolite collection from U2OS MYC-ER cells was performed in an identical fashion to that described in Fig 8A. Rhythmicity was assessed by ECHO for both MYC-OFF and MYC-ON, with metabolites with a 20–28 hour period and with BH.Adj.P.Value < 0.05 deemed rhythmic. These metabolites were sorted by phase and are presented in a heatmap for MYC-OFF. For MYC-ON, the same metabolites that are rhythmic in MYC-OFF are presented in the same order, but with MYC-ON values instead. B. Metabolites from Replicate B deemed to be oscillating by ECHO for MYC-OFF are graphed, with dots representing the relative abundance values, and lines indicating the fitted oscillation curves as calculated by ECHO. Metabolites with a green border are oscillatory in both MYC-OFF and MYC-ON. C, D. For Replicate A (C) from Fig 8, and Replicate B (D), metabolites deemed oscillatory from MYC-ON are shown, sorted by phase. For MYC-OFF, the same metabolites that are rhythmic in MYC-ON are presented, but with MYC-OFF values instead. (TIFF) [file pgen.1010904.s009.tiff]

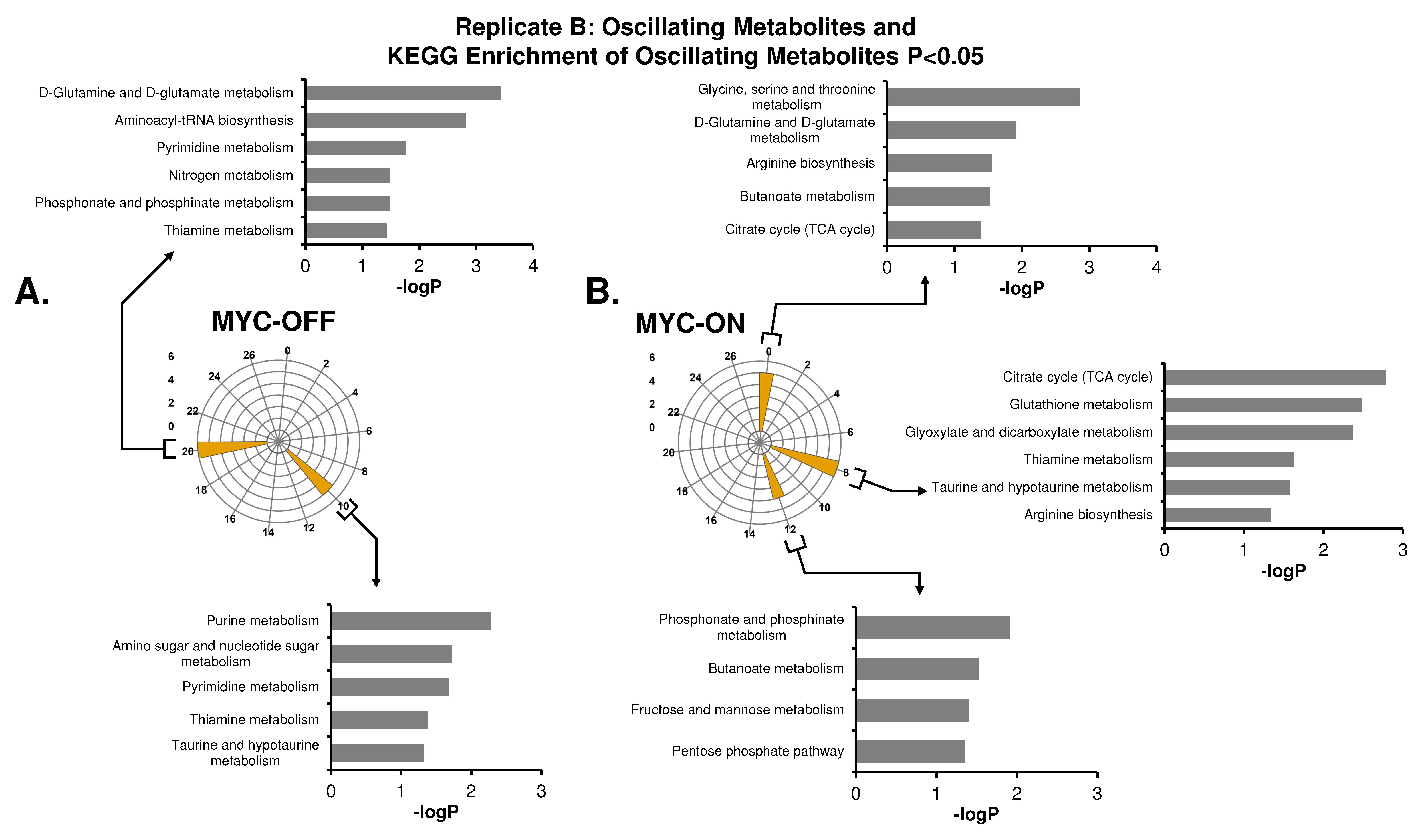

Supplement: S9 Fig — A,B. KEGG enrichment analysis was performed on metabolites that peaked in the indicated phases from Replicate B in MYC-OFF (A) or MYC-ON (B) conditions, and significantly enriched pathways were graphed on a polar histogram. For each histogram, the scale is on the left side. Pathways with a p < 0.05 were deemed significant and are graphed. (TIFF) [file pgen.1010904.s010.tiff]
