# Supplementary material for: MYC disrupts transcriptional and metabolic circadian oscillations in cancer and promotes enhanced biosynthesis
Source: PLoS Genet. 2023 Aug 28;19(8):e1010904. doi: 10.1371/journal.pgen.1010904 (PMC10491404; doi:10.1371/journal.pgen.1010904)
Supplement: Attachment — Submitted filename: 133174_1_rebuttal_2750937_ryfftn.pdf [file pgen.1010904.s011.pdf]

Full Revision

Manuscript number: RC-2023-01817
Corresponding author(s): Altman, Brian; Dang, Chi; Weljie, Aalim

[Please use this template only if the submitted manuscript should be considered by the affiliate journal as a full revision in response to the points raised by the reviewers.

If you wish to submit a preliminary revision with a revision plan, please use our "Revision Plan" template. ***It is important to use the appropriate template to clearly inform the editors of your intentions.****]*

1. General Statements [optional]

We thank all Reviewers for their detailed and helpful comments and suggestions for this manuscript. The overall goal of this study is to interrogate which transcriptional and metabolic pathways lose oscillation when MYC is amplified or activated. We have now added additional replicates to our RNA-sequencing and nutrient transporter expression analyses, and have demonstrated that MYC disrupts oscillation of metabolic and biosynthetic gene expression, nutrient transporter oscillation, and metabolite pathways. On the suggestion of Reviewer #3, we have also strengthened this work by directly contrasting the transcriptional oscillations we observe in cancer cells with well-established primary cell models of transcriptional oscillation, MEFs and macrophages. We have carefully responded to each comment and have noted which Figures or lines in the manuscript address each comment. We hope that our revised manuscript is now suitable for publication.

Data availability: All input and processed data relating to this Manuscript have been uploaded to FigShare. A persistent DOI has been generated and will be Published upon acceptance. For Reviewers, please use this private link to review the data. We ask that the Reviewers keep these data and this link confidential until acceptance of the work.
https://figshare.com/s/4058d4cba6b212645264

2. Point-by-point description of the revisions

This section is mandatory. Please insert a point-by-point reply describing the revisions that were already carried out and included in the transferred manuscript.

Reviewer 1

This is an interesting paper from the Altman, Weljie and Dang labs that furthers their previous publications looking at the effect of "oncogenic" Myc levels have on circadian gene expression. They provide compelling data that in neuroblastoma and osteosarcoma models of Myc amplification circadian gene expression and metabolite fluctuation are lost. The data is convincing and comprehensive and should be of broad general interest. There are a few major issues that need to be addressed.

Review COMMONS

(Significance (Required)):

The Myc oncogene is dysregulated in many cancer types, so there is considerable interest in the mechanisms that underpin its function as a transforming oncogene. This group of authors has previously described that Myc can disrupt circadian gene expression, which is linked to several types of cancer. This paper extends the authors previously findings by performing careful timed RNA-seq analysis and metabolomic analysis. The work is well done, and the findings justify the conclusions. This paper should be of interest to those who study Myc, circadian gene expression and cancer. Two key limitations are noted: 1) the cells that are analyzed are grown entirely in vitro in serum and nutrient replete media, 2) there is no direct evidence that the blockage of circadian gene expression by Myc is important for Myc-dependent transformation, although it seems likely. These two limitations do not detract from the significance of the manuscript.

My expertise is in cancer-centric gene regulatory mechanisms

We thank Reviewer #1 for finding the data compelling and comprehensive, and for suggesting key experiments and revisions. We also thank the Reviewer for noting that this will be of interest to those who study MYC and cancer (noted in the Significance section below). We have addressed all suggestions below.

1) Is there a control (done here or in prior literature) showing that simply adding tam to cells doesn't change circadian gene expression?

We previously performed this experiment in our 2015 *Cell Metabolism* paper (Altman and Hsieh, et al). In particular, by using either cells that did not express MYC-ER, or a control mutant MYC-ER that lacks transcriptional activity, we found the 4OHT did not blunt clock function in U2OS cells. This is now discussed in Lines 120-121 of the revised manuscript.

2) All of the experiments rely on Myc:ER fusions. The authors should mine other datasets to determine of systems that rely on conditional expression of wt Myc drive a similar loss of circadian gene expression.

This is an important point: the work in this manuscript focuses on cancers with endogenous levels of MYC where MYC-ER fusions drive changes in gene expression. In our previous works, we tested systems such as the Reviewer suggested, where endogenous MYC was conditionally overexpressed (Altman and Hsieh et al. *Cell Metabolism* 2015, Altman et al *Nature Communications* 2017). In these systems (liver cancer and Burkitt's Lymphoma), elevated MYC also leads to dysfunction of circadian gene expression and oscillation. We reference this work on lines 123-126. In addition, two papers from the laboratory of Michael Brunner (Shostak et al *Nature Communications 2016,* Shostak et al *Nature Communications* 2017) used a tetracycline-inducible model of wild-type MYC in U2OS instead of MYC-ER. Their results largely mirrored

our own, where BMAL1 was suppressed and molecular circadian oscillation was disrupted. These two papers are cited extensively throughout manuscript.

3) The authors argue Myc:ER fusions mimic Myc amplification which is common in cancer and they discuss several previous papers that at least correlate the presence of amplified Myc with loss of circadian gene expression. It should be possible to test whether reduction of Myc by knockdown or using a Myc inhibitor restores circadian gene expression in a cell line known to be Myc amplified. This need not be an exhaustive RNA-seq experiment, but looking at a handful of circadian genes using a qPCR approach would be informative.

Thank you for the suggestion. Our previous work in Burkitt's Lymphoma and liver cancer cell lines, as well as analysis of data from primary liver cancer, drew on MYC Tet-OFF systems, where amplified MYC is suppressed by addition of tetracycline or doxycycline. In these experiments, the 'control' is amplified MYC, and the 'experimental' condition is tet- or dox-treated cells where MYC has been suppressed. In all cases, suppressing MYC led to predictable changes in circadian gene expression (suppression of REV-ERBα, upregulation of BMAL1, etc).

To support these findings, upon the suggestion of the Reviewer, we identified a recent study where the PC3 prostate cancer cell line, known to harbor highly elevated endogenous MYC levels, was treated with the new generation MYC inhibitor MYCi361. We downloaded their RNA-sequencing data and performed differential expression analysis, and showed that several circadian genes were significantly altered upon treatment with the inhibitor, including BMAL1, PER2, and the REV-ERB genes. This is now included as Supplemental Figure S1C, and the text for this is on lines 126-136.

4) The data pretty clearly shows that metabolites lose their periodicity MycON cells. Can these be linked back to loss of circadian expression of specific genes in those metabolic pathways? If so, are genes direct Myc transcriptional targets in other studies?

Thank you for bringing this up. We now have computational evidence from multiple replicate circadian time-series experiments that MYC disrupts oscillation of the LAT1 amino acid transporter across multiple cell lines (Figure 6), and that MYC upregulates LAT1 and 4F2hc protein and mRNA (Supplemental Figure 6 and not shown). Indeed, LAT1 is known to be a direct MYC target, which is now mentioned on lines 493-499 of the discussion.

In U2OS, where we performed our metabolomics studies, while LAT1 oscillates at the protein level, we saw less evidence of metabolic program oscillation at the transcriptional level, which we acknowledge on lines 274-277. This may be due to the fact that not all metabolic and protein oscillations arise solely from transcriptional oscillation, which we mention on lines 48-49 of the introduction, and revisit in lines 430-432 of the discussion. Nonetheless, our findings that MYC disrupts oscillation of nutrient transporters and metabolites fits in with the overall theme of this manuscript that MYC disrupts circadian control of metabolism.

5) The finding Myc activation "releases" metabolic and biosynthetic pathways from circadian control implies that this must have something to do with Myc-dependent transformation. A priori, it is not obvious why this should be the case. Do metabolic precursors and biosynthetic molecules become periodically limiting when their levels oscillate in MycOFF cells? In MycOn cells do the non-oscillating metabolites, provide a growth advantage? This is a difficult question to address and one that is certainly beyond the scope of this manuscript. The authors should address this issue in their discussion.

Thank you for the suggestion to discuss this idea in more detail. While we propose the hypothesis that circadian metabolic oscillations are limiting for tumor cells, testing this directly is indeed outside the scope of this current study. We address this issue on lines 506-518 of the discussion, where we contrast our hypothesis with the idea that alternate metabolic oscillations (those tied to cell cycle or faster-than-circadian) may arise in the absence of circadian control.

Minor points
1) phrase "for the first time" is used multiple times in the discussion. Gets a bit redundant (and loses impact). Consider revising.

Thank you for this suggestion. We have revised our Discussion section accordingly.

2) In figure 4, the periodicity in expression of the proteins in figure 4 is fairly clear, but it might be beneficial to bracket (or denote in some other way) the circadian fluctuation in expression.

In response to other Reviewer comments, we performed multiple replicates of the nutrient transporter protein expression time-series, quantified protein, and calculated circadian oscillations. This is now presented in the new **Figure 6**.

Referees cross-commenting

I had not considered the important points raised by reviewer 3. The authors definitely need to address the concern over replicates and whether the gene expression of truly rhythmic. If not, this seems like a fatal flaw in the MS.

We have carefully addressed both of these concerns by adding replicates to our RNA-sequencing and protein expression assays, and contrasting our gene expression oscillation findings with those in established primary cell models (**Supplemental Figure 2A**). Please see the Response to Reviewer 3 for more detail.

Reviewer 2
(Significance (Required)):
Using the circadian synchronized cancer cell lines, DeRollo and colleagues characterized the MYC oncoprotein role in metabolic role through the circadian clock disruption. Authors found

that forced activation of MYC disrupts up to 85% of genes oscillation particularly nutrient transporter glycosylation and amino acid metabolism. This work addresses important questions in the circadian clock and cancer field through the oncogene activation, and the manuscript is well-written. However, there are a few concerns that should be addressed to improve the manuscript quality.

We thank Reviewer #2 for their detailed and astute suggestions on demonstrating the degree of MYC overexpression and the synchronization / entrainment of our cells, as well as the suggestion to add and quantify multiple replicates. We also appreciate the Reviewer's comments in the Significance section that the manuscript addresses important questions in the field and is well-written. We have individually addressed each comment below and made several revisions and additions in response to them.

Major comments:

1) The 3 cell lines used in this paper, what is the expression levels of MYC protein under -OFF and -ON conditions? It is important to demonstrate this information through the western blot data. Since the 4-hydroxy tamoxifen was used to activate MYC, what is the vehicle/control for MYC OFF cells? Otherwise, it will be difficult to assess with everything observed on this manuscript under MYC-ON could be due to 4-hydroxy tamoxifen treatment.

Thank you for this important consideration. We have clarified in the manuscript that the MYC-ER system is constitutively expressed, and when cells are treated with 4OHT, MYC-ER is activated and translocates to the nucleus, while MYC-OFF control cells are treated with ethanol as a vehicle (Lines 100-105). In response to the suggestion to quantify the degree of overexpression, we have also added new experiments to quantify the degree of MYC-ER overexpression, in **Supplemental Figure 1A and 1B**. Finally, we previously showed in our 2015 *Cell Metabolism* paper (Altman and Hsieh, et al) that 4OHT does not affect the molecular clock on its own. In particular, by using either cells that did not express MYC-ER, or a control mutant MYC-ER that lacks transcriptional activity, we found the 4OHT did not blunt clock function in U2OS cells. This is discussed in Lines 120-121 of the revised manuscript.

2) In Fig. 1A, it is crucial to demonstrate that the circadian synchronization protocol is working by performing statistical analysis with at least 3 biological replicates. This should be performed by either cosinor analysis and/or JTK cycle analysis of all the canonical clock genes including BMAL1 (ARNTL), CLOCK, CRY1, CRY2, PER1, PER2, DBP and NR1D1. This reviewer would like to see both transcripts (qPCR) and protein levels (western blot data) of those clock genes expression pattern. Without these results, rest of the data will be hard to conclude the connection with the circadian/molecular clock.

Thank you for bringing of the need for quantitation of circadian transcripts. In the new **Figure 1**, we have quantified and performed ECHO analysis (which is a parametric method of oscillation analysis used through the manuscript) on several circadian transcripts. We chose to specifically show the same n=2 input RNA that we used for RNA-seq for each cell lines. Additionally, our

updated RNA-sequencing and analysis of oscillating genes in MYC-OFF shows that CRY2, PER2, PER3 oscillate in all three cell lines (**Figure 2B**). These findings agree with extensive literature by us and others that the molecular circadian clock is functional after dexamethasone entrainment in U2OS, SHEP, and SKNAS: Baggs et al *Plos Biology* 2009, Zhang et al *Cell* 2009, Hughes et al *Plos Genetics* 2009, Altman and Hsieh et al. *Cell Metabolism* 2015, Altman et al *Nature Communications* 2017, Shostak et al *Nature Communications 2016,* Shostak et al *Nature Communications* 2017.

3) In Fig. 5A-C: It is important to repeat this western blot experiment at least 3 times and have the quantitation to demonstrate the circadian rhythmicity significance by probing to majority of the canonical clock proteins as discussed above.

Thank you for this suggestion. The western blot experiments have now been repeated and have n=3-4 replicates, have been quantified, and oscillation assessed. We also quantified and plotted the oscillation of BMAL1 and REV-ERBα as comparisons. This is in the new **Figure 6**.

Minor Comments:

1) For all of the western blot images, authors need to show the molecular weight of the corresponding protein bands detected on the blots.

All raw western blot images, including molecular weights, will either be published as Supplemental Material or on FigShare (with a persistent doi), depending on the preference of the Journal. A private link is available for Reviewers with all the relevant background data, including westerns with molecular weights: https://figshare.com/s/4058d4cba6b212645264

2) As a proof of concept, it would be interesting if knockout/knockout the MYC gene in these cell lines and look for the expression pattern of canonical clock gene expression levels to see whether it will help enhancing circadian rhythmicity. If authors cannot perform this experiment, it is important to address under discussion.

Thank you for this idea. Reviewer 1 had a similar idea / comment. We addressed it through previous studies, and a new analysis of MYC-high PC3 prostate cancer cells treated with the MYC inhibitor MYCi361 (**Supplemental Figure 1C**). See response to Review 1 Major Point 3 for more details.

3) It is not discussed, how many biological replicates were used for RNA-seq analysis?

In the revised manuscript, each RNA-sequencing experiment is performed from n=2 biological replicates and n=13-14 time points per replicate.

Referees cross-commenting

Review COMMONS

I completely agree with reviewer #3. In fact, I have raised the similar points in my major comments #2 and # 3.

As discussed below, we have responded to Reviewer comments with more replicates and new analyses of oscillating genes and proteins.

Reviewer 3

Review of "MYC Disrupts Transcriptional and Metabolic Circadian Oscillations in Cancer and Promotes Enhanced Biosynthesis"
DeRollo et al. attempt to find commonalities in how MYC affects transcriptional and metabolic programming by examining MYC-switchable U2OS, SHEP, and SKNAS cell lines. They claim that oncogenic MYC both represses transcriptional oscillation of many genes and supports rhythmic expression of other genes. They use RNA-Seq and UPLC-MS/MS with the appropriate bioinformatics analyses. In some cases, they employ qPCR and immunoblotting. In the three different cell lines, they observed that MYC either statically upregulates or downregulates oscillatory genes.

(Significance (Required)):

Myc is known to interfere with the rhythmic expression of core circadian clock genes. Myc seems to do this in order to rewire control-clock expression programs in favor of cell growth and proliferation.
DeRollo et al intended to investigate which clock-controlled expression programs are deregulated by MYC. For this purpose, they investigated three cell lines. Unfortunately, it seems that clock-controlled genes are not really express with a (sufficiently) substantial amplitude in these cultured cells. It is therefore not possible to distinguish by RNA-seq the truly rhythmic genes from false positives. Therefore, it is not possible to reliably determine which metabolic rhythmic programs are deregulated by MYC.

We thank Reviewer #3 for their important observations and suggestions with regards to the number of replicates employed, and the confidence in the oscillations we observe. We have responded to these comments in a detailed fashion by adding replicates to our RNA-sequencing and immunoblot, and by comparing the oscillations in our cell lines to established primary models of circadian oscillation to determine the amplitudes of oscillations we observed.

As it stands, the work has technical and conceptual weaknesses.
First, it is not clear how many replications the authors performed for RNA seq. For U2OS, this is explicitly stated. Replicate 1: four-hours sampling, ribosomal RNA depleted; replicate 2: two-hours sampling, polyA+ RNA. There do not appear to be replicates for the other two cell lines?

Thank you for this observation. In the initial manuscript submission, we used n=2 replicates for U2OS and SHEP, and n=1 replicate for SKNAS (apologies for this not being clear). In response

to your comment and others, we added a new biological replicate for RNA-sequencing for SKNAS, so each cell line now has n=2 replicates. This allowed us to more confidently identify oscillating genes across biological replicates for each cell line.

U2OS cells are widely used in circadian research. These cells rhythmically express clock genes with a decent amplitude, which the authors confirmed by qPCR. However, the clock-controlled genes are generally expressed at a very low amplitude (in the range of standard deviation of RNA-Seq). It is therefore extremely difficult to identify and distinguish them from nonrhythmic genes by RNA seq. The fact that the authors find approximately the same number of rhythmic genes in MYC-OFF (Fig. 1B) as in MYC-ON (Supplemental Fig. 1B) and no overlap between the three cell lines tells that most of the genes shown in the heat maps are not truly rhythmic. Rather, they appear to represent those genes that are called rhythmic because a cosine wave happens to fit the data (better than a line). I suspect that true replicates (which are missing) would also show little or no overlap because most genes in these cells are probably not really rhythmic with any significant amplitude (and why would they be under constant conditions in a Petri dish?).
Thus, if there are no rhythmic clock-controlled genes that can be clearly distinguished from non-rhythmic genes, there is no way to tell which rhythms are attenuated by MYC (apart from the core clock genes shown in Fig. 1A), or to identify potentially rhythmic pathways.

Thank you for bringing up this important point about the confidence in the rhythmicity of genes examined. As is correctly noted and as brought up in response to Reviewer #2, cell lines such as U2OS, SKNAS, and SHEP have extensively been used for molecular clock studies (see Reviewer 2 Major Point 2). We also note that each cell line is now n=2 biological replicates, so all oscillating transcripts represent genes that were oscillating in both biological replicates. We take seriously the concern that the oscillating transcripts are not truly rhythmic, or of insufficient amplitude to be biologically significant. We employed a published algorithm, ECHO (De Los Santos et al, *Bioinformatics* 2019), which uses a conservative parametric approach to determine oscillations from sequencing data, and filters out genes that are too lowly expressed for oscillations to be determined, and those where a sharp increase or decrease in gene expression would preclude determining oscillations.

To directly test the strength of our observed oscillations in MYC-OFF conditions, we downloaded and analyzed time-series RNA-sequencing data from two entrained primary cell models that are known to have robust transcriptional circadian oscillations: MEFs and macrophages. These two datasets were analyzed in the same fashion as our cell lines, using the ECHO parametric algorithm, and we plotted the median amplitude of oscillation for all transcripts that had significant circadian oscillation (**Supplemental Figure 2A**). We found that the median amplitude of oscillation was within the same range as those from primary cells: SHEP cells showed nearly identical median amplitude to MEFs, while U2OS and SKNAS had slightly higher median amplitudes than macrophages. These suggest that the oscillating transcripts we observe and measure represent true oscillations above background noise that

Review COMMONS

are similar to those observed in primary cell models where transcriptomic oscillation has been extensively studied.

With regards to oscillations observed in MYC-ON: we would first like to note that in some cases, there are fewer oscillating genes, especially in SKNAS, where there are less than half the number of oscillating genes in MYC-ON as compared to MYC-OFF. Nonetheless, the observation of emergent oscillations in MYC-ON cells is interesting, and we devote a paragraph to this in the Discussion (lines 457-482). We note that major perturbations to the molecular clock, such as DKO of REV-ERBα and β, result in emergent oscillations in the liver (Guan D et al, *Science*, 2020), and speculate that oscillations observed in MYC-ON may be from residual activity of CLOCK and BMAL1, which may occupy new sites when MYC is overexpressed.

If there are no strong rhythmic clock-controlled genes, there are probably no strong rhythms in clock-controlled metabolism. Indeed, the authors found no overlap in rhythmic metabolites.

Because metabolomics is far less sensitive than RNA-sequencing, we performed KEGG enrichment analysis on oscillating metabolites from both our replicates. We found that in MYC-OFF, the enriched metabolic pathways were *identical* in timing and identity of enriched pathways (**Figure 8, Supplementary Figure 9**), while these were quite divergent in MYC-ON. Thus, we concluded that common oscillating metabolic pathways in the absence of MYC are altered or disrupted by MYC activation.
